# Long-term survival in metastatic abdominal Ewing sarcoma with gastric serosal involvement following first-line irinotecan plus cisplatin: a case report

**DOI:** 10.3389/fonc.2026.1766821

**Published:** 2026-04-30

**Authors:** Lianhua Ji, Yutong Li, Xiaofeng Chen, Yuyang Liu, Wei Tao, Guoqun Wang, Tingyun Cui, Yi Chen, Hui Sun, Nanyuan Jiang, Kangxin Wang

**Affiliations:** 1 Department of Oncology, Nanjing Pukou People’s Hospital, Liangjiang Hospital, Southeast University, Nanjing, Jiangsu, China; 2 School of Stomatology, Nanjing Medical University, Nanjing, China; 3 Department of Oncology, The First Affiliated Hospital of Nanjing Medical University, Nanjing, China; 4 Gastric Cancer Center, The First Affiliated Hospital of Nanjing Medical University, Nanjing, China; 5 Jiangsu Key Lab of Cancer Biomarkers, Prevention and Treatment, Collaborative Innovation Center for Personalized Cancer Medicine, Nanjing Medical University, Nanjing, China; 6 College of Clinical Medicine, Xuzhou Medical University, Xuzhou, China; 7 Department of Radiation Oncology,Nanjing Pukou People’s Hospital, Liangjiang Hospital, Southeast University, Nanjing, Jiangsu, China

**Keywords:** case report, cisplatin, Ewing sarcoma, first-line therapy, irinotecan, long-term survival

## Abstract

**Background:**

Abdominal primary Ewing sarcoma (ES) is extremely rare and carries a dismal prognosis when metastatic. First-line therapy typically involves multi-agent regimens like VDC/IE, with irinotecan-based combinations reserved for relapsed/refractory disease. This case is unique due to the achievement of long-term survival using first-line irinotecan and cisplatin in a patient with aggressive abdominal ES.

**Case presentation:**

A 25-year-old female presented initially with hematochezia, and a huge abdominal mass after postoperative pathology confirmed a diagnosis of high-grade ES/PNET with rapid postoperative progression with new splenic metastases and regional lymph node involvement (no pulmonary metastases). Owing to factors related to treatment tolerance, she received an individualized treatment approach consisting of irinotecan plus cisplatin combined chemotherapy. Serial tumor response assessments demonstrated continuous reduction, culminating in a complete response (CR) that has been maintained to the present. The progression-free survival (PFS) has reached 76 months, representing a significant achievement in terms of long-term, high-quality survival.

**Conclusion:**

This case suggests that for selected patients with advanced ES, a regimen of irinotecan plus cisplatin may be considered as a potential first-line option. Larger studies are needed to validate these findings.

## Introduction

Ewing sarcoma/primitive neuroectodermal tumor (ES/PNET) is an extremely rare, highly malignant small round cell neoplasm that predominantly affects pediatric and adolescent populations, with a peak incidence at approximately 15 years of age and a male-to-female ratio of 3:2 (1). ES/PNET can arise in any anatomical location; approximately 80% of cases originate in the skeletal system, commonly involving the pelvis, vertebral column, ribs, and long bones of the extremities. Extraskeletal ES accounts for approximately 20% of cases, occurs more frequently in adults, and often involves paraspinal regions and soft tissues of the thoracic wall (2).Although approximately 80% of Ewing sarcoma family tumors (ESFT) arise from bone, extraskeletal ESFT are not uncommon, with one series reporting 22 out of 73 cases (30%) as extraosseous (17). Rarely, ES may develop within visceral organs, with primary gastric involvement being exceptionally uncommon. The tumor is characterized by aggressive behavior, rapid progression, and a tendency for early metastasis, leading to an overall poor prognosis. At initial diagnosis, approximately 20–25% of patients present with distant metastatic disease, most commonly involving the lungs, bones, and bone marrow.

ES is a highly chemosensitive malignancy; however, the 5-year survival rate is only approximately 10% with local therapies alone, such as surgery or radiotherapy. The addition of chemotherapy has significantly improved patient outcomes. Based on the 2025 NCCN guidelines and the latest evidence (3), the treatment of advanced ES recommends multi-agent combination regimens, specifically VIDE (vincristine, ifosfamide, doxorubicin, etoposide) or VDC/IE (vincristine, doxorubicin, cyclophosphamide alternating with ifosfamide and etoposide). These regimens are administered as induction chemotherapy for a minimum of nine weeks (Level I evidence).Regimens that incorporate topoisomerase I inhibitors (e.g., topotecan or irinotecan) in combination with cyclophosphamide and temozolomide have demonstrated favorable response rates in patients with recurrent or refractory osteosarcoma (4–6). Despite recent advances achieved through multidisciplinary approaches (including chemotherapy, surgery, and radiotherapy), which have improved the 5-year survival rate to approximately 70%, the relapse rate remains high. This is particularly true for metastatic cases, which carry a markedly poorer prognosis (7), thereby underscoring the urgent need for better therapies.

Herein, we presented a remarkable case of advanced abdominal ES with multi-organ involvement that achieved a durable complete remission exceeding 76 months following first-line chemotherapy with irinotecan and cisplatin, a regimen not commonly employed upfront. This case provides valuable insights for the management of rare, recurrent, and refractory tumors and is presented to aid clinical decision-making in oncology treatment planning. Meanwhile, this case also highlights a potential alternative therapeutic pathway for selected patients and underscores the need for personalized treatment strategies.

## Case presentation

**Figure 1:**
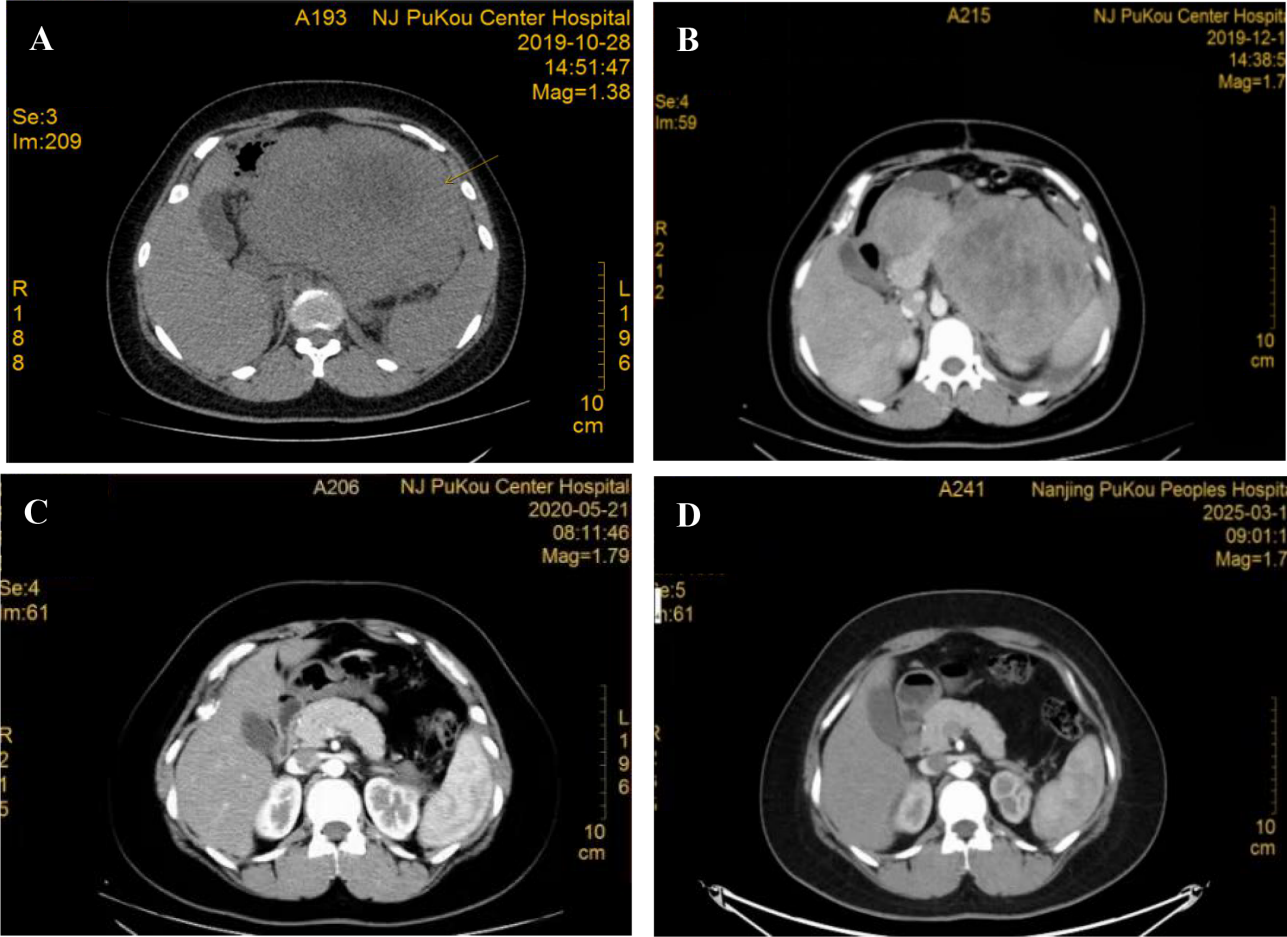
Changes of radiography at different period of treatment. **(A)** CT scan performed at baseline (October 28, 2019); **(B)** CT scan performed at one month after surgery when early progression with new metastases (December 13, 2019); **(C)** CT scan assessment of best response of CR (May 21, 2020); **(D)** Recent CT scan assessment of CR (March 11, 2025);.

**Figure 2:**
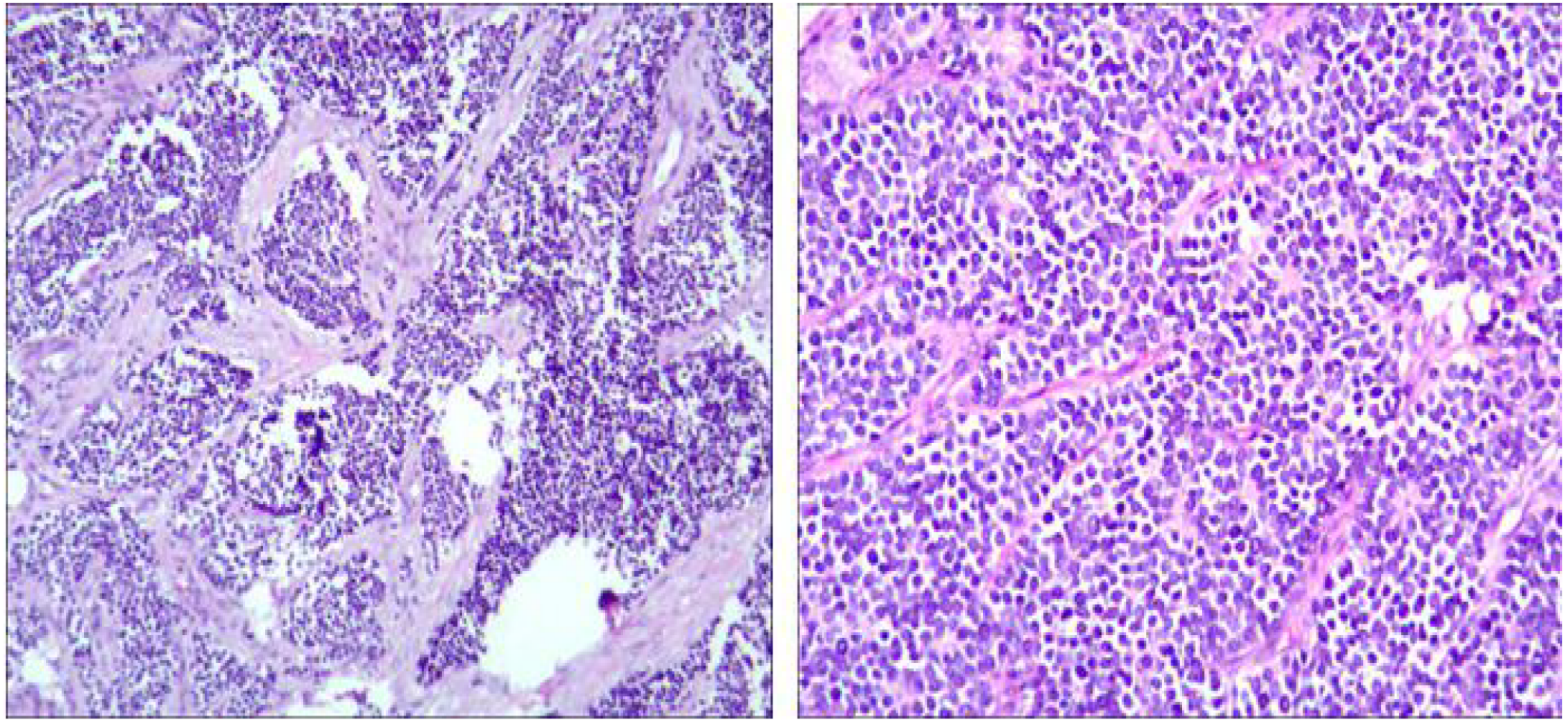
Postoperative pathological section.

A 25-year-old Chinese female with no significant past medical history initially presented in October 2019 with hematochezia. She denied any history of smoking, alcohol use, or family history of cancer. A gastroscopy performed on October 26, 2019, revealed gastric fundal varices and chronic superficial gastritis but no mass or ulcer. An abdominal contrast-enhanced CT scan on October 28, 2019, showed a large mass occupying the stomach, measuring approximately 18 × 12 cm, initially suggestive of lymphoma or a stromal tumor (**Figure 1A**). On October 30, 2019, the patient underwent resection of the intra-abdominal lesion. Intraoperative findings revealed a large tumor (approx. 20 × 15 cm) along the greater curvature of the stomach, adherent to the gastric body, transverse mesocolon, and lateral peritoneum.Importantly, the surgeon performed tumor resection with repair of the gastric serosal defect, rather than gastric partial resection, indicating that the tumor likely originated from the perigastric soft tissue, with secondary invasion and adhesion to the gastric serosa. Postoperative histopathological examination confirmed an abdominal high-grade small round cell malignant tumor involving the pancreatic invasion. Consistent with ES/PNET, The complete immunohistochemical panel revealed: CD99 (3+, diffuse membranous), vimentin (+), CD117 (+), Syn (+), CD56 (focal+), CK (perinuclear dot-like), and Ki-67 (approximately 70%). Negative markers included: CgA, NSE, Desmin, SMA, MyoD-1, CD31, CD34, Dog-1, EMA, CK8/18, LCA, CD20, CD30, CD79α, CD3, CD5, S-100, SOX-10, HMB45, PLAP, SALL4, and GPC-3. These findings, together with the characteristic morphology of small round blue cells, confirmed the diagnosis of PNET/Ewing sarcoma. Of note, EWSR1 rearrangement analysis (FISH/PCR) was not performed as this molecular test was not routinely available at our institution at the time of diagnosis (2019) (**Figure 2**). Based on the medical history and diagnostic findings, the patient was initially diagnosed with locally advanced abdominal ES. A postoperative CT scan on November 6, 2019 (one week after surgery) showed residual disease in the left upper abdomen but a normal spleen with no evidence of metastases. Therefore, the initial clinical stage was cT4N1M0.

However, the tumor demonstrated rapid postoperative progression. A restaging CT scan on December 13, 2019 (six weeks after surgery) revealed new splenic metastases, enlarged abdominal lymph nodes, and left kidney invasion (**Figure 1B**). Chest CT showed no pulmonary metastases. Systemic therapy was therefore initiated for metastatic disease (Stage IV). Considering the anticipated poor tolerability to prolonged, intensive multi-agent chemotherapy and the need to prevent tumor lysis syndrome, an alternative first-line regimen was chosen after discussion with the patient and obtaining informed consent.

The treatment protocol was as follows:

1. Systemic Chemotherapy (Dec 2019 - May 2020): The patient received six cycles of irinotecan (240 mg, day 1) plus cisplatin (40 mg, days 1-2) via intravenous infusion, repeated every 21 days.
2. Maintenance Therapy (May 2020 - ongoing): After achieving complete response (CR), maintenance therapy with single-agent irinotecan was started. The dose was initially reduced to 200 mg every 28 days and continued for two years. Given sustained remission, the dose was further reduced to 180 mg, administered intermittently every three months, and this maintenance therapy has been ongoing.

Radiotherapy was not administered at any point during the patient’s treatment course.

To facilitate understanding, a timeline summarizing the patient’s clinical course is presented in **Table 1**. This visual representation outlines key treatment milestones, evaluations, and the development of tumor response.

**Table 1:** Timeline of the patient’s clinical course.

Date	Event
Oct 2019	Initial Presentation & Diagnosis: Presented with hematochezia. CT scan revealed a large abdominal mass. Surgical resection confirmed ES. Postoperative CT (Nov 6, 2019) showed residual disease but normal spleen (no metastases).
Dec 2019	Postoperative Progression: Rapid Postoperative Progression: CT scan (Dec 13, 2019) revealed new splenic metastases, enlarged lymph nodes, and left kidney invasion. No pulmonary metastases. Stage IV metastatic disease confirmed.
Dec 2019 - May 2020	First-Line Chemotherapy: Received 6 cycles of IP regimen (Irinotecan + Cisplatin). Achieved CR by end of treatment.
May 2020 - Jun 2022	Monotherapy Maintenance: Received reduced-dose Irinotecan monthly. Sustained CR.
Jun 2022 - Present	Reduced-dose Intermittent Monotherapy: Dose reduced, interval extended to every 3 months. Continued CR with PFS of 76 months (as of April 2026).

Response evaluation, including radiography and tumor markers, were carried out termly according to the Response Evaluation Criteria in Solid Tumors version 1.1. Within the chemotherapy period, the patient achieved a best response of CR (**Figure 1C**) and long-term duration of response. Also, the treatment was overall well-tolerated. The patient experienced grade 1–2 nausea and vomiting, and grade II myelosuppression (leukopenia and anemia). Notably, she did not experience diarrhea. Renal function remained normal throughout treatment, and no ototoxicity was observed, which was managed with supportive care (granulocyte colony-stimulating factor, erythropoietin, and hematopoietic supplements), with no other severe adverse events. Comfortingly, throughout the subsequent dose reduction maintenance treatment stage, the disease status was still consistently assessed as CR according to the most recent CT scan on March 11, 2025 (**Figure 1D**), has consistently confirmed the absence of disease recurrence. As of April 2026, the patient’s progression-free survival (PFS) was 76 months and is expected to extend further, representing an exceptional long-term, high-quality survival outcome.

## Discussion

Among undifferentiated small round cell sarcomas, ES is generally associated with a relatively favorable prognosis, demonstrating an overall 5-year survival rate of approximately 70%. However, the prognosis is strongly influenced by the disease stage at initial diagnosis. Patients with localized disease have a survival rate exceeding 70%, whereas those with recurrent or metastatic disease face a significantly poorer prognosis, with survival rates dropping to less than 30% (8). Local or distant recurrence is common and carries a poor prognosis. Additionally, the pattern of metastasis affects outcomes: patients with isolated pulmonary metastases have a more favorable prognosis than those with widespread dissemination, and central axial lesions (e.g., pelvis or spine) confer a poorer prognosis compared to lesions in the limbs. Furthermore, studies have identified several adverse prognostic factors, including a tumor volume greater than 100 mL, diagnosis in adulthood (age >18 years), and elevated serum lactate dehydrogenase levels, all of which are associated with worsened survival (9, 10).

For advanced Ewing sarcoma, the NCCN guidelines recommend first-line chemotherapy using the VDC/IE regimen, an alternating schedule of vincristine, doxorubicin, cyclophosphamide, and ifosfamide. For second-line and subsequent therapies, multimodal approaches that combine chemotherapy with local treatments such as surgery or radiotherapy are recommended. In relapsed or refractory osteosarcoma, regimens containing topoisomerase I inhibitors (e.g., topotecan or irinotecan) in combination with cyclophosphamide and temozolomide have demonstrated favorable response rates (4–6).Irinotecan-based regimens, as well as the related topoisomerase I inhibitor topotecan, have demonstrated considerable activity in recurrent/refractory Ewing sarcoma family tumors (15, 16). Xu J, Xie L, et al. evaluated the antitumor activity of irinotecan-based regimens in a phase II clinical trial for recurrent or refractory ES. They found that irinotecan-based chemotherapy demonstrated the most favorable outcomes among the protocols evaluated. Specifically, irinotecan in combination with agents such as temozolomide or cyclophosphamide achieved the highest objective response rate (ORR) of 36%–40%, a disease control rate (DCR) of approximately 60%, and a median PFS of 8.1 months, thereby outperforming other chemotherapeutic combinations (11). Asaftei SD, Puma N, et al. assessed the efficacy and tolerability of the combination of temozolomide and irinotecan (TEMIRI) as first-line therapy for previously untreated patients with primary disseminated Ewing sarcoma (PDMES) (12). Early TEMIRI regimens demonstrated promising activity, achieving an ORR of 59% and a disease progression rate of only 9%. The 3-year event-free survival (EFS) and overall survival (OS) rates were 21% (95% CI: 6–35) and 36% (95% CI: 18–54), respectively. In the phase II Euro-EWING study, irinotecan was used as frontline neoadjuvant therapy (13) and was associated with an ORR of 24% and a disease progression rate of 29%. Based on these results, single-agent irinotecan is not recommended for patients with ES. Additionally, in a study of PDMES patients who received two cycles of cisplatin as neoadjuvant therapy, the ORR was only 8%, and no significant survival benefit was observed (14). Therefore, irinotecan-based chemotherapy is primarily used as a second- or third-line treatment for relapsed or metastatic ES and is not currently a standard first-line therapy.

This report detailed a case of high-grade ES/PNET with postoperative rapid progression with new splenic metastases and regional lymph node involvement, thereby necessitating a highly individualized treatment approach to optimize the therapeutic outcome. In accordance with clinical guidelines, systemic multi-agent chemotherapy is the recommended first-line treatment. However, due to the prolonged induction period and anticipated poor tolerability associated with multi-agent regimens, coupled with the need to prevent treatment-related tumor lysis syndrome, a first-line regimen consisting of irinotecan and cisplatin was administered. During therapy, a continuous reduction in tumor burden was observed, culminating in a complete remission that has been maintained. The PFS of 76 months represents a significant achievement, reflecting long-term, high-quality survival. This notable clinical benefit could be attributed to the tumor’s biological characteristics, including sensitivity to topoisomerase I inhibitors or platinum-based agents, as well as potential molecular alterations such as a high methylation status or other changes that confer sensitivity to irinotecan, similar to mechanisms observed in some small cell carcinomas. The patient received prolonged maintenance irinotecan therapy after achieving CR. While maintenance chemotherapy has shown benefit in other sarcomas (e.g., rhabdomyosarcoma), its role in Ewing sarcoma remains undefined. Therefore, it cannot be concluded that the exceptional long-term survival is attributable to maintenance therapy rather than the initial response to irinotecan-cisplatin. Given the favorable clinical response observed in this case, further clinical investigations are warranted to assess whether the irinotecan plus cisplatin regimen may serve as a potential first-line option for selected patients with advanced Ewing sarcoma.

## Limitations

Several limitations of this report should be acknowledged. First, as a single case report, the findings cannot be generalized to all patients with Ewing sarcoma. Second, molecular confirmation via EWSR1 rearrangement analysis (FISH or PCR) was not performed as this test was not routinely available at our institution at the time of diagnosis (2019). The diagnosis was based on characteristic morphology, a comprehensive IHC panel (including CD99 3+ and FLI-1 nuclear positivity), and expert pathology review. Third, the patient received maintenance irinotecan therapy following the initial response; therefore, the long-term survival cannot be definitively attributed solely to the first-line irinotecan-cisplatin regimen. Larger prospective studies are needed to validate these findings and identify predictive biomarkers.

## Conclusion

This case involves a high-grade small round cell malignant tumor with an abdominal cavity origin, which is an exceedingly rare entity. The tumor exhibited early pancreatic invasion at diagnosis and demonstrated rapid progression to multi-organ involvement shortly after surgery, indicative of a highly aggressive phenotype. Considering factors including treatment tolerability, an individualized treatment approach with irinotecan and cisplatin was employed at our center. This regimen successfully induced CR, demonstrating significant efficacy. Given the patient’s stable condition, a maintenance strategy of low-dose, intermittent chemotherapy was adopted to balance efficacy and quality of life. This approach has achieved sustained tumor remission and enabled long-term, high-quality survival, with the potential for further survival extension. This case suggests that the combination of irinotecan with platinum-based chemotherapy may be considered a promising first-line treatment option for selected patients with ES. However, further exploration of the tumor’s immune microenvironment and molecular genetic profile is needed to identify predictive biomarkers for treatment response. Furthermore, validation through large-scale, prospective clinical trials is required to confirm the efficacy of this regimen and to define the patient population most likely to benefit.

## Data Availability

The original contributions presented in the study are included in the article/Supplementary Material. Further inquiries can be directed to the corresponding authors.
